# Exosomal miR-155 from gastric cancer induces cancer-associated cachexia by suppressing adipogenesis and promoting brown adipose differentiation *via* C/EPBβ

**DOI:** 10.20892/j.issn.2095-3941.2021.0220

**Published:** 2022-02-19

**Authors:** Ying Liu, Meng Wang, Ting Deng, Rui Liu, Tao Ning, Ming Bai, Guoguang Ying, Haiyang Zhang, Yi Ba

**Affiliations:** 1 Tianjin Medical University Cancer Institute and Hospital, National Clinical Research Center for Cancer, Tianjin’s Clinical Research Center for Cancer, Key Laboratory of Cancer Prevention and Therapy, Tianjin 300060, China

**Keywords:** Exosomes, adipose mesenchymal stem cells, miR-155, cachexia, gastric cancer

## Abstract

**Objective:**

The aim of this research was to identify whether exosomes were involved in impairing adipogenesis in cancer-associated cachexia (CAC) by detecting the adipodifferentiation capacity and the expressions of adipogenic proteins in gastric cancer (GC)-associated adipocytes.

**Methods:**

Western blotting and RT-PCR were used to investigate the expressions of C/EPBβ, C/EPBα, PPARγ, and UCP1 in adipose mesenchymal stem cells (A-MSCs) to evaluate the function of exosomal miR-155. BALB/c nude mice were intravenously injected *in vivo* with GC exosomes with different levels of miR-155 to determine changes in adipodifferentiation of A-MSCs.

**Results:**

Exosomes derived from GC cells suppressed adipogenesis in A-MSCs as characterized by decreased lipid droplets. Similarly, A-MSCs co-cultured with GC exosomes exhibited increased ATP production through brown adipose differentiation characterized by highly dense mitochondria and enhanced UCP1 expression (*P* < 0.05). Mechanistically, exosomal miR-155 secreted from GC cells suppressed adipogenesis and promoted brown adipose differentiation by targeting C/EPBβ, accompanied by downregulated C/EPBα and PPARγ and upregulated UCP1 (*P* < 0.05). Moreover, overexpression of miR-155 in GC exosomes improved CAC *in vivo*, which was characterized by fat loss, suppressed expressions of C/EPBβ, C/EPBα, and PPARγ in A-MSCs, and high expression of UCP1 (*P* < 0.05). Decreasing the level of miR-155 in injected GC exosomes abrogated the improved CAC effects.

**Conclusions:**

GC exosomal miR-155 suppressed adipogenesis and enhanced brown adipose differentiation in A-MSCs by targeting C/EPBβ of A-MSCs, which played a crucial role in CAC.

## Introduction

Cancer-associated cachexia (CAC) is a metabolic disorder characterized by progressive loss of body fat and skeletal muscle wasting^1^, and is a common occurrence in gastric cancer (GC), which accounts for 20% of cancer-related deaths^2^. Recent studies and clinical trials of CAC treatments have focused on changes in adipose tissue, especially increased lipolysis in CAC^3^. However, the role of adipose mesenchymal stem cells (A-MSCs) in CAC is poorly understood. Thus, detailed studies are required to identify the changes in adipogenesis during CAC, which have been identified as key hallmarks of the balance with lipolysis.

Adipocytes are developed from A-MSCs, which are similar to multipotent adult stem cells and have the capacity to differentiate into several cell types^4^. The renewal of adipocytes relies on orchestrated activation of transcription factors that induce the differentiation of A-MSCs to mature adipocytes^5^. A-MSCs are therefore a perfect cell model to study adipogenesis at the level of stem cells. Members of the CCAAT/enhancer-binding protein (C/EBP) family and the preceding peroxisome proliferator-activated receptor (PPAR) family are the 2 most critical transcription factor families associated with adipogenic-specific programs, with C/EPBβ playing an essential role^6–8^. During A-MSC adipodifferentiation, activated C/EBPβ triggers transcription of *C/EPB*α and *PPAR*γ genes, which in turn coordinately activates proteins, such as fatty acid-binding proteins and fatty acid transport proteins (FATPs) responsible for the differentiation of lipid droplet (LD)-containing cells into terminal adipocytes^9,10^.

Adipose tissue is generally comprised of 2 different types, namely, white adipose tissue (WAT) and brown adipose tissue (BAT). These tissues usually perform different biological functions. WAT is characterized by LDs and few mitochondria, and contributes to storage of energy. BAT is characterized by significant uncoupling protein 1 (UCP1) expression and contains small lipid droplets and numerous mitochondria to produce heat^11,12^. Brown adipose differentiation contributes to significant increases in total energy expenditure and aggravated CAC. However, the direct molecular mechanism leading to brown adipose differentiation is not well understood. Exosomes are microvesicles with diameters of 40–160 nm that are released by most cell types^13^. Studies have shown that exosomes play an important role in intercellular communication. Exosomes contain a variety of RNAs, proteins, and lipids. A recent report in *Nature Reviews* showed that extracellular vesicle packing cargo reaches subpopulations of muscle cells and mediates cachexia^14^. Additionally, pancreatic cancer exosomes induce lipolysis by activating p38 and ERK1/2 MAPKs^15^. Previous studies in our laboratory have shown that GC exosomal ciRS-133s are involved in WAT browning and play a key role in CAC^16^. However, the effects of GC exosomes on adipogenesis have not been elucidated.

In the current study, we showed that miR-155 was upregulated in the plasma and A-MSCs from GC patients, and was negatively correlated with LD levels. Additionally, an *In vitro* study demonstrated that the inhibition of LD formation and promotion of brown adipose differentiation in A-MSCs were caused by exosomal miR-155 secreted from GC cells. Moreover, further studies revealed that miR-155 directly targeted the 3′-UTR of *C/EPB*β, which finally blocked C/EPBα and PPARγ proteins, and increased UCP1 expression. An *in vivo* analysis of the levels of C/EPBβ in MSCs from mouse inguinal adipose tissues (mA-MSCs) as well as C/EPBα, PPARγ, and UCP1 expression in mA-MSCs demonstrated that knockdown of miR-155 in GC exosomes significantly blocked brown adipose differentiation and LD impairment of A-MSCs caused by GC. Herein, the specific targeting of exosomal miR-155 from GC on C/EPBβ was confirmed to be involved in CAC, characterized by decreasing LDs and increased brown adipose differentiation in A-MSCs.

## Materials and methods

### Cell culture

Adipose tissues were obtained from healthy donors undergoing liposuction and from the peritoneum of GC patients according to procedures approved by the Ethics Committee of Tianjin Medical University Cancer Institute and Hospital (Approval No. EK2017012). A-MSCs were isolated and expanded as previously described^7^. The cells were incubated in DMEM/F-12 (Gibco, Gaithersburg, MD, USA) supplemented with 10% fetal bovine serum (FBS; Gibco), 1% penicillin, and streptomycin (Solarbio, Beijing, China), 2 mmol/L glutamine and 10 ng/mL epidermal growth factor (PeproTech, Cranbury, NJ, USA). To induce A-MSC adipogenesis, A-MSCs remained in the adipogenic differentiation medium containing DMEM/F-12, 1 µM dexamethasone, 0.5 mM 3-isobutyl-1-methylxanthine, 0.5 µg/mL insulin, 0.5 mM isobutyl methylxanthine, and 10% FBS.

SGC7901 and MGC803 (human gastric adenocarcinoma cell lines) cells were purchased from the cell bank of the Chinese Academy of Sciences (Shanghai, China) and then cultured in DMEM (Gibco) supplemented with 10% FBS.

### Exosome isolation and identification

Exosomes in media were isolated according to a previous report^16^. GC cell culture media were collected at 300 × *g* and 3,000 × *g* to eliminate cells and debris. Next, the supernatant was centrifuged at 110,000 × *g* for 70 min at 4 °C to obtain exosomes.

The exosomes were fixed overnight in 2.5% glutaraldehyde at pH 7.2 at 4 °C. The prepared samples were washed in phosphate-buffered saline (PBS) 3 times (5 min each time) and fixed in 1% osmium tetroxide for 60 min at room temperature. Then, the sample blocks were prepared by embedding the samples in 10% gelatin, fixing them in glutaraldehyde at 4 °C, and then cutting the samples into several blocks (< 1 mm). Dehydration of the sample blocks was performed for 10 min at each step using increasing concentrations of alcohol (30%, 50%, 70%, 90%, 95%, and 100% × 3, 10 min, each step). Using propylene oxide to exchange pure alcohol, the specimens were then infiltrated with increasing concentrations (25%, 50%, 75%, and 100%) of Quetol-812 epoxy resin (Nisshin, Tokyo, Japan) mixed with propylene oxide, with at least 3 h per step. The samples were then embedded in pure, fresh Quetol-812 epoxy resin and polymerized at 35 °C for 12 h, 45 °C for 12 h, and 60 °C for 24 h. Ultrathin sections (100 nm) were cut using a Leica UC6 ultramicrotome (Leica, Wetzlar, Germany) and then stained with uranyl acetate for 10 min, then with lead citrate for 5 min at room temperature before observation using an FEI Tecnai T20 transmission electron microscope (Philips, Amsterdam, The Netherlands).

Western blotting was performed to detect exosome-specific markers, including TSG101 and CD63. The mean diameters of exosomes were measured using a NanoSight NS 300 system (NanoSight Technology, Malvern, UK).

### RNA isolation and quantitative RT-PCR

Total RNA was isolated from cultured cells, exosomes, and tissues using TRIzol Reagent (Invitrogen, Carlsbad, CA, USA) according to the manufacturer’s instructions. The cDNA was obtained by using avian myeloblastosis virus reverse transcriptase (TaKaRa, Shiga, Japan). Quantitative analysis of miR-155 was detected using TaqMan miRNA probes (Applied Biosystems, Foster City, CA, USA) and normalized to U6 snRNA expression. The mRNA expression levels of C/EPBβ, C/EPBα, and PPARγ were determined with a SYBR Green PCR Master Mix (Qiagen, Hilden, Germany) using ABI PRISM (Applied Biosystems). The mRNA expression was normalized to β-Actin. All reactions were performed at least in triplicate. Relative levels of genes were calculated using the 2-ΔCT method. The primer sequences were as follows:

 5′-GGGCCCTGAATCGCTTA A-3′ (C/EPBβ, sense);

 5′-ATCAACAGCAACAAGCCCG-3′ (C/EPBβ, anti-sense);

 5′-GAAGTTGGTGGAGCTGTCGG-3′ (C/EPBα, sense);

 5′-TGAGGTATGGGTCGTTGGGA-3′ (C/EPBα, anti-sense);

 5′-AGCCTCATGAAGAGCCTTCCA-3′ (PPARγ, sense);

 5′-ACCCTTGCATAATTCACAAGC-3′ (PPARγ, anti-sense);

 5′-GGCTGTGCTATCCCTGTACG-3′ (β-actin, sense); and

 5′-CTTGATCTTCATTGTGCTGGGTG-3′ (β-actin, anti-sense).

### Western blotting

Total protein was extracted using RIPA buffer, and the protein concentration was determined using a BCA Protein Assay kit (Thermo Fisher Scientific, Waltham, MA, USA). Protein (30 µg) of each sample was loaded onto a 10% SDS-PAGE gel, resolved, then transferred to a polyvinylidene fluoride membrane (Millipore, Burlington, MA, USA). After blocking with 5% bovine serum albumin at room temperature for 1 h, the membranes were incubated with primary antibodies (1:1,000 dilution) at 4 °C overnight. After the membranes were incubated with the corresponding secondary antibodies (1:5,000 dilution) for 1 h at room temperature, the blots were detected using an enhanced chemiluminescence solution (Invitrogen) and visualized with a ImageQuantLAS-4010 (GE Healthcare, Chicago, IL, USA).

### Oil Red O staining

The cells were gently washed 3 times with PBS and then fixed with 4% formaldehyde for 10 min at room temperature. After fixation, the cells were treated with filtered 0.25% Oil Red O solution for 40 min. Red-stained lipid droplets in the cells were observed and photographed using light microscopy.

### Luciferase assay

The miRNA target prediction and analysis were performed with algorithms from TargetScan (http://www.targetscan.org/vert_72/), PicTar(https://pictar.mdc-berlin.de/), and miRanda (http://miranda.org.uk/).

The reporter plasmid, p-MIR-C/EPBβ, was designed by Genescript (Nanjing, China) and contained the predicted miR-155 binding site. Parts of the wild-type and mutated 3′-UTR of *C/EPB*β were cloned into the firefly luciferase reporter. For the luciferase reporter assays, 200 ng firefly luciferase reporter plasmid, 200 ng β-galactosidase vector, and equal doses (200 pmol) of mimics, inhibitors, or scrambled negative control RNAs were transfected into prepared cells. At 24 h after transfection, the luciferase activity of cells was analyzed using the Dual Luciferase Assay Kit (Promega, Madison, WI, USA) according to the manufacturer’s instructions.

### Immunofluorescence

Cells were cultured in 12-well plates with slides. At the harvest, the cells were first fixed with 4% paraformaldehyde and then permeabilized with 0.2% Triton X-100 for 10 min, followed by incubation for 1 h. The cells were then incubated with UCP1 antibodies (1:100; Abcam, Cambridge, MA, USA) at 4 °C overnight and goat anti-rabbit IgG (Invitrogen) for 1 h in the dark at room temperature. The 4′,6-diamidino-2-phenylindole (Solarbio) was used for nuclear staining. For confocal microscopy, a Nikon C2 Plus confocal microscope (Nikon, Tokyo, Japan) was used.

### Immunohistochemistry

The inguinal adipose tissues were fixed in 10% formaldehyde, embedded in paraffin, sectioned, and then stained with anti-UCP1 antibodies (Abcam). For confocal microscopy, an Olympus BX43 microscope (Olympus, Tokyo, Japan) was used.

### Establishment of GC cachexia in nude mice

According to procedures approved by the Ethics Committee of Tianjin Medical University Cancer Institute and Hospital (NSFC-AE-2020002), SGC7901 cell exosomes with different levels of miR-155 were intravenously injected into nude mice. The groups are described in **Figure 5A**. Eighteen days later, the plasma and inguinal adipose tissues were harvested.

### Statistical analysis

All experiments were performed in triplicate, and the results are presented as the mean value ± standard deviation. The data were statistically analyzed using Student’s *t*-test using SPSS statistical software for Windows (SPSS, Chicago, IL, USA), with *P < 0.05 indicating statistical significance, **indicating *P* < 0.01, and ***indicating *P* < 0.001.

## Results

### Adipocyte differentiation of A-MSCs from GC patients is inhibited and negatively correlated with miR-155

**Figure 1:**
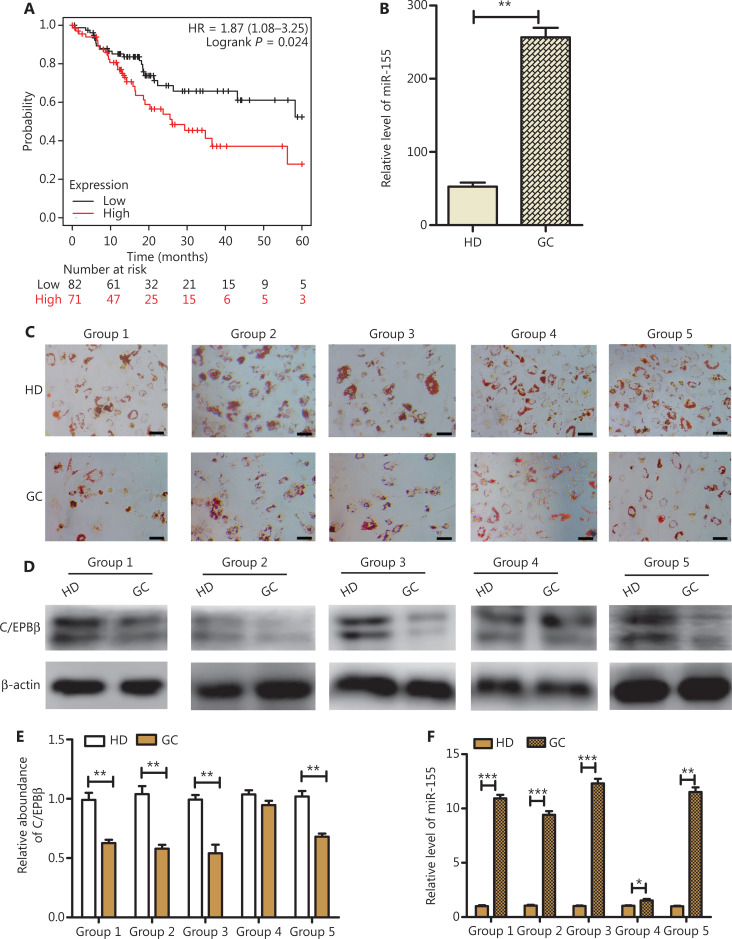
The miR-155 showed a negative relationship with adipocyte differentiation of adipose mesenchymal stem cells (A-MSCs) in gastric cancer (GC) patients. (A) Relationship between miR-155 and GC survival from the tumor database. (B) Relative levels of miR-155 in serum from GC patients and healthy donors using RT-PCR (*N* = 150). (C) Oil Red O staining analysis of the adipo-differentiation capacity of A-MSCs from GC patients and healthy donors (*N* = 5, scale bar = 100 μm). (D, E) Western blot analysis of C/EBPβ expressions in A-MSCs in GC patients (*N* = 5). (F) Relative levels of miR-155 in A-MSCs from GC patients and healthy donors (*N* = 5). *P < 0.05; **P < 0.01; ***P < 0.001.

We initially analyzed the survival of GC patients using a tumor database (http://kmplot.com), which revealed that a high miR-155 expression level was correlated with a shorter overall survival (*P* < 0.05). The values of miR-155 therefore indicated poor prognoses of GC patients (**Figure 1A**). The levels of miR-155 in the serum of GC patients and healthy donors were then measured. A high level of miR-155 expression was detected in the serum of GC patients (*P* < 0.01) (**Figure 1B**). We also detected LDs in A-MSCs isolated from peritoneal adipose tissues of GC patients (GC group) and A-MSCs from subcutaneous adipose tissues of healthy donors (HD group) as normal controls. As shown by Oil Red O staining, the accumulation of LDs in A-MSCs isolated from the peritoneum of GC patients decreased (**Figure 1C**). As a crucial transcription factor of the early adipocyte differentiation stage, C/EBPβ was significantly decreased in A-MSCs isolated from GC patients (*P* < 0.01) (**Figure 1D and 1E**). We performed RT-PCR to detect the expression levels of miR-155; the results indicated that miR-155 in A-MSCs was increased in the GC group, when compared with the HD group (*P* < 0.05) (**Figure 1F**). In conclusion, the above results suggested that miR-155 promoted GC progression and was negatively correlated with the adipogenic capacity of A-MSCs.

### Identification and characterization of A-MSCs and GC exosomes

The morphology of A-MSCs was similar to that of fibroblasts (**Supplementary Figure S1A**), and LD formation was observed after culturing in adipogenic differentiation medium (**Supplementary Figure S1B and S1C**). A-MSCs were positive for CD44, CD73, CD90, CD105, CD166, and HLA-ABC, but negative for CD31, CD133, CD14, CD34, CD45, and HLA-DR (**Supplementary Figure S1D**). In our preliminary experiments, transmission electron microcopy showed that SGC7901 exosomes exhibited a round-shaped morphology (**Supplementary Figure S1E**) and had a size ranging from 50 to 160 nm (**Supplementary Figure S1G**). In addition, TSG101 and CD63 were highly expressed by SGC7901 and MGC803 exosomes (**Supplementary Figure S1F**). Exosomes stained with PKH26, for general cell membrane labeling, were added to the A-MSC medium, and phagocytosis was recorded using confocal microscopy at 6 h after co-culturing. Over 80% of the A-MSCs exhibited PKH67 staining through the red fluorescence (**Supplementary Figure S1H**). Taken together, our results showed that A-MSCs and exosomes with high purity were successfully isolated, and that exosomes could be taken up by A-MSCs.

### GC cell-derived exosomes inhibit adipogenesis of A-MSCs

**Figure 2:**
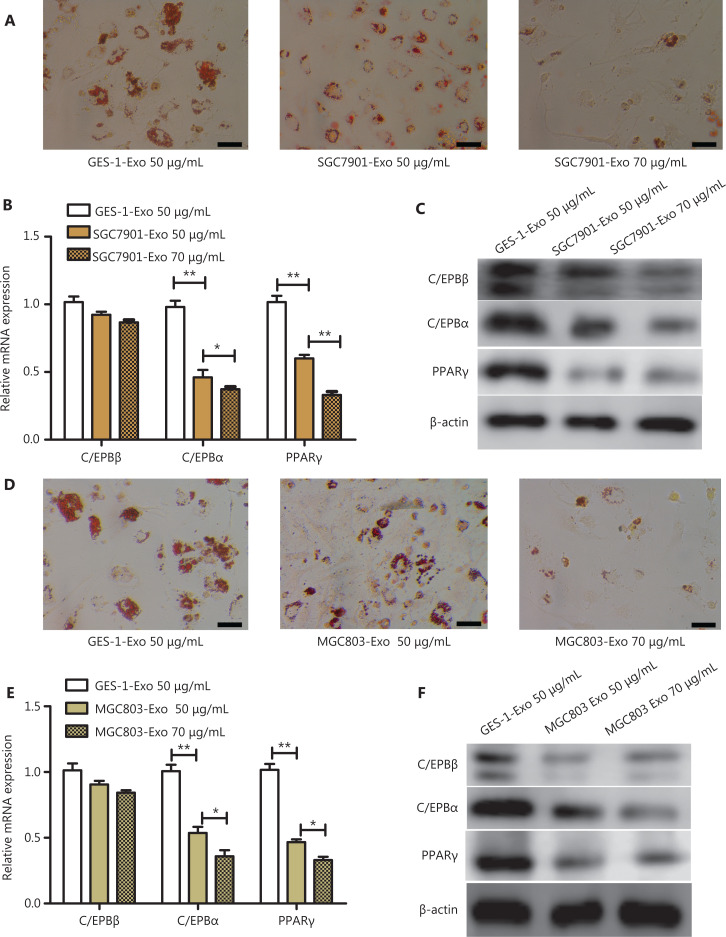
Gastric cancer exosomes inhibited adipocyte differentiation of adipose mesenchymal stem cells (A-MSCs). (A) Oil Red O staining was performed to visualize lipid droplet accumulation in A-MSCs treated with GES-1-Exo or SGC7901-Exo. (*N* = 3, scale bar = 100 μm). (B) RT-PCR analysis of adipogenic-specific genes (*C/EBP*β, *C/EBP*α, and *PPAR*γ were normalized to β-actin) in the groups described above (*N* = 3). (C) Western blot analysis of C/EBPβ, C/EBPα, and PPARγ in the groups described above (*N* = 3). (D) Oil Red O staining was performed to detect lipid droplet accumulation in A-MSCs treated with GES-1-Exo or MGC803-Exo. (*N* = 3, scale bar = 100 μm). (E) RT-PCR analysis of adipogenic-specific genes and (F) western blot analysis of the expression of C/EBPβ, C/EBPα, and PPARγ in the groups described above (*N* = 3). *P < 0.05; **P < 0.01.

Because SGC7901 exosomes and MGC803 exosomes could be taken up by A-MSCs, we hypothesized that the adipogenic differentiation of A-MSCs was changed by GC exosomes. A-MSCs were treated with different doses of SGC7901 exosomes (SGC7901-Exo) and MGC803 exosomes (MGC803-Exo) (50 µg/mL and 70 µg/mL, respectively) in adipogenic medium for 5 days. The SGC7901-Exo treatment significantly decreased the number of LDs in A-MSCs as the concentration of exosomes increased, when compared with the GES-1 exosomes [(GES-1-Exo) 50 µg/mL] treatment group (**Figure 2A**). SGC7901-Exo also decreased the mRNA expression levels of the adipogenic transcription factors C/EBPα and PPARγ (*P* < 0.05) but had no effect on the level of C/EBPβ mRNA (*P* > 0.05) (**Figure 2B**). Moreover, protein expressions of C/EBPβ, C/EBPα, and PPARγ in A-MSCs cultured in adipogenic medium were inhibited by SGC7901-Exo (*P* < 0.05) (**Figure 2C**). Densitometry analyses of western blot images is shown in **Supplementary Figure S2A**. Similarly, MGC803-Exo impaired LD formation (**Figure 2D**), decreased the mRNA expression levels of the adipogenic transcription factors, C/EBPα and PPARγ (*P* < 0.05) (**Figure 2E**), and decreased the protein expressions of C/EBPβ, C/EBPα, and PPARγ in A-MSCs (*P* < 0.05) (**Figure 2F**) during adipogenesis. The densitometry analyses of the western blot images are shown in **Supplementary Figure S2B**. These results suggested that GC exosomes acted as a negative regulator of adipogenesis in A-MSCs.

### GC cell-derived exosomes promote browning differentiation of A-MSCs

**Figure 3:**
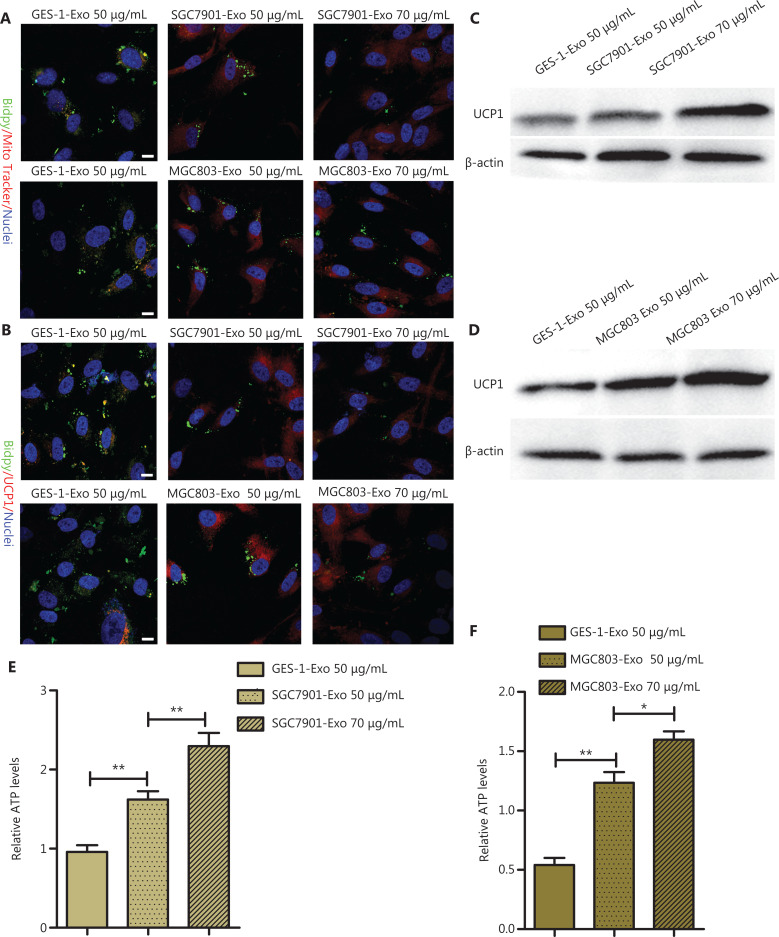
Gastric cancer cell-derived exosomes promoted browning of white adipose tissue. (A) Representative images of MitoTracker staining (red labeling) with BODIPY staining for lipid droplets (green labeling) (*N* = 3, scale bar = 10 μm). (B) Immunofluorescence assay for UCP1 (red labeling) with BODIPY staining for lipid droplets (green labeling) (*N* = 3, scale bar = 10 μm). (C) Western blot analysis of UCP1 expressions in adipose mesenchymal stem cells (A-MSCs) treated with GES-1-Exo and SGC7901-Exo. (D) Western blot analysis of UCP1 expression in A-MSCs treated with GES-1-Exo and MGC803-Exo (*N* = 3). (E) Relative ATP levels of A-MSCs treated with GES-1-Exo and SGC7901-Exo (*N* = 3). (F) Relative ATP levels of A-MSCs treated with GES-1-Exo and MGC803-Exo (*N* = 3). *P < 0.05; **P < 0.01.

To analyze the browning traits influenced by GC exosomes, A-MSCs were co-cultured with SGC7901-Exo and MGC803-Exo, and the phenotypic, molecular, and metabolic responses of A-MSCs were assessed. Compared to the GES-1-Exo co-cultured, A-MSCs showed increased brown adipocyte features, such as increased mitochondrial abundance and UCP1 expression, after treatment with CG exosomes (SGC7901-Exo and MGC803-Exo) (**Figure 3A and 3B**). Western blot analysis also indicated that UCP1 expression in A-MSCs cultured in adipogenic medium was upregulated by GC exosomes (*P* < 0.01) (**Figure 3C and 3D**). The densitometry analysis for the western blot images is shown in **Supplementary Figure S3C and S3D**. After treatment with GC exosomes, the relative levels of ATP in A-MSCs were enhanced (*P* < 0.05) (**Figure 3E and 3F**). Together, these results revealed that GC exosomes promoted browning differentiation in A-MSCs.

### GC exosomal miR-155 directly targets C/EBPβ in A-MSCs

**Figure 4:**
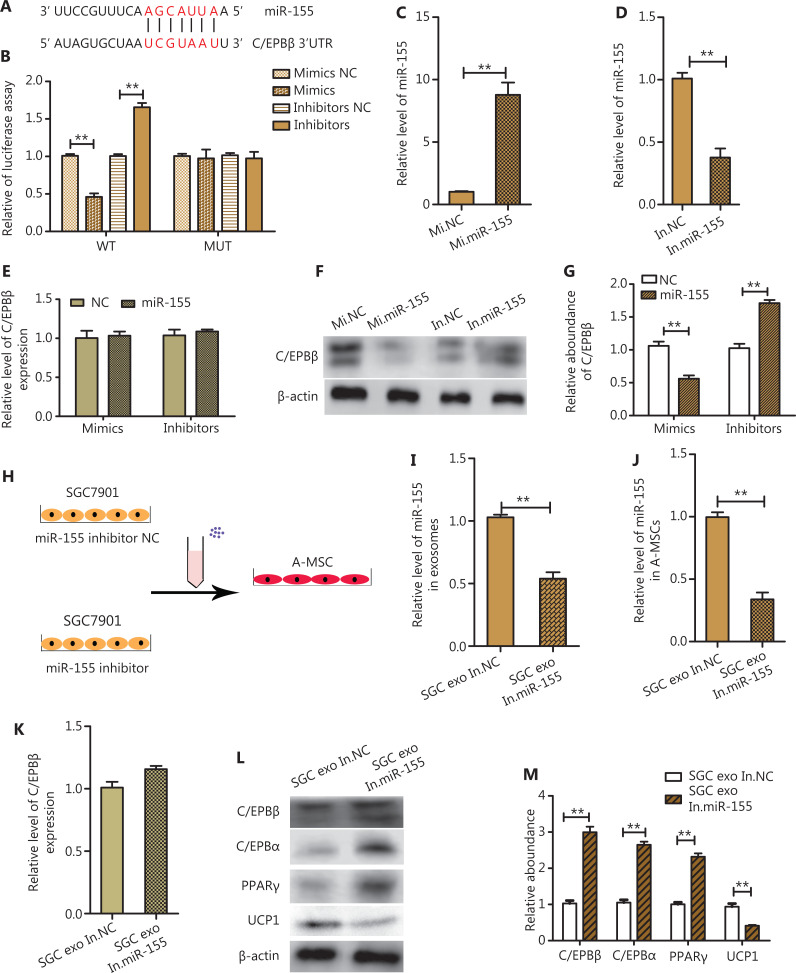
Gastric cancer-exosome-miR-155 targeting C/EBPβ in adipose mesenchymal stem cells (A-MSCs). (A) Predicted binding sites for miR-155 in the 3′-UTR of C/EBPβ mRNA. (B) Direct recognition of C/EBPβ by miR-155. Firefly luciferase reporters containing either the wild-type (WT) or mutant (MUT) C/EBPβ 3′-UTR sequence, miR-155 mimics, miR-155 inhibitors and the corresponding normal control were co-transfected into A-MSCs. The relative luciferase levels were detected (*N* = 3). (C–D) RT-PCR analysis of miR-155 levels in A-MSCs transfected with normal control mimics (Mi NC), miR-155 mimics (Mi miR-155), normal control inhibitors (In NC), and miR-155 inhibitors (In miR-155) (*N* = 3). (E) RT-PCR analysis of C/EBPβ mRNA levels in the groups described above (*N* = 3). (F–G) C/EBPβ expression in the groups described above (*N* = 3). (H) Schematic description of the experimental design. Isolation of exosomes after inhibiting miR-155 levels in SGC7901 cells and normal controls and adding to A-MSCs. (I) RT-PCR assay of miR-155 levels in SGC exo In.miR-155 or SGC exo In.NC (*N* = 3). (J) RT-PCR assay of miR-155 levels in A-MSCs treated with SGC exo In.miR-155 or SGC exo In.NC (*N* = 3). (K) C/EBPβ mRNA levels in A-MSCs pretreated with different exosomes were detected by RT-PCR (*N* = 3). (L–M) C/EBPβ, C/EBPα, PPARγ, and UCP1 expressions in A-MSCs treated with SGC exo In.miR-155 and SGC exo In.NC analyzed by western blotting (*N* = 3). **P < 0.01.

**Figure 5:**
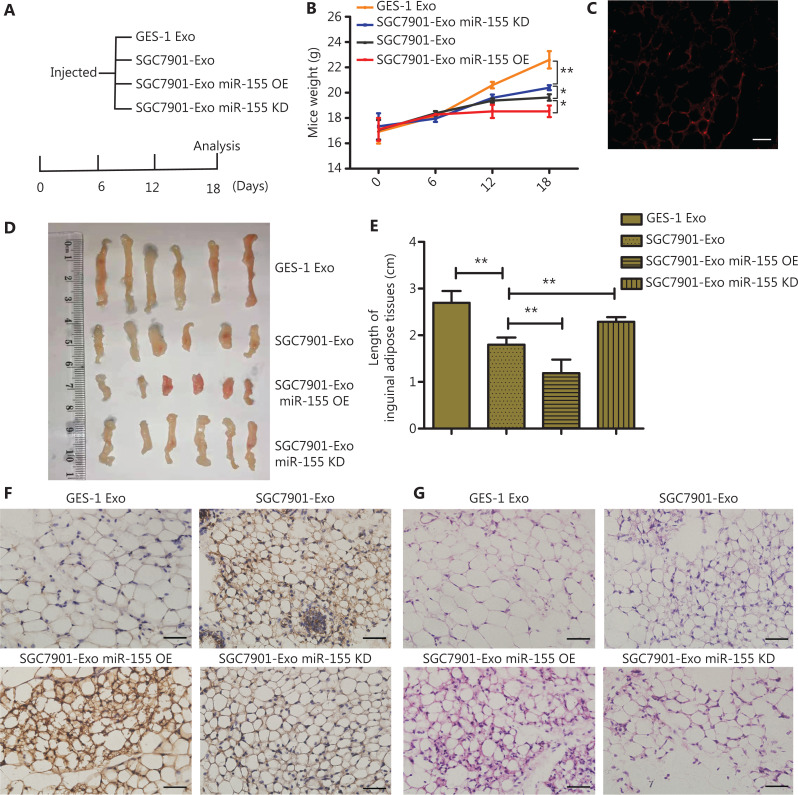
Influence of exosomal miR-155 in gastric cancer on loss of adipose tissue *in vivo*. (A) Flow chart depicting the *in vivo* experimental design. (B) Weight change of the mice (*N* = 3). (C) Morphology of the inguinal adipose tissue after the mice were injected with PKH67-labeled SGC7901 exosomes (scale bar = 100 μm). (D–E) Images of inguinal adipose tissue and analysis of the length (*N* = 6). (F) Expression of UCP1 in inguinal adipose tissue was identified by immunohistochemistry (*N* = 3, scale bar = 100 μm). (G) Representative images from hematoxylin and eosin (H&E)-stained inguinal adipose tissue (*N* = 3, scale bar = 100 μm). *P < 0.05; **P < 0.01.

The miR-155 binding sites in the 3′-UTR of C/EBPβ mRNA are shown in **Figure 4A**. Transfection of miR-155 mimics significantly reduced luciferase activities, whereas luciferase levels were relatively enhanced by miR-155 inhibitors (*P* < 0.01). However, the inhibitory activity was lost when the binding sites were mutated (**Figure 4B**). These results indicated that miR-155 suppressed C/EBPβ expression by binding to the C/EBPβ 3′-UTR in A-MSCs. Additionally, to further determine the influence of miR-155 on adipogenic differentiation of A-MSCs, normal control mimics (Mi NC), miR-155 mimics (Mi miR-155), normal control inhibitors (In NC), and miR-155 inhibitors (In miR-155) were directly transfected into A-MSCs. As shown in **Figure 4C and 4D**, RT-PCR was used to analyze the levels of miR-155 in the above groups. After culturing in adipogenic differentiation medium for 5 days, western blotting was used to assess the expression level of C/EBPβ. The expression of C/EBPβ in A-MSCs was much lower in the miR-155 mimic group than in the normal control mimic group (*P* < 0.01), while the expression of C/EBPβ was significantly enhanced in A-MSCs transfected with miR-155 inhibitors compared with the normal control inhibitors group (*P* < 0.01). However, there was no significant change in C/EBPβ mRNA (**Figures 4E and 5F and 5G**). Moreover, we also found that miR-155 was highly expressed in exosomes derived from SGC7901 cells and MGC803 cells (*P* < 0.01) (**Supplementary Figure S4**). These results indicated that C/EBPβ was a downstream target of miR-155.

### Downregulated miR-155 in GC exosomes rescues browning differentiation and poor adipogenesis in A-MSCs

To verify the function of miR-155 in GC exosomes, SGC7901 cells were transfected with miR-155 inhibitors or normal control inhibitors, and exosomes from each group (SGC exo In.miR-155 or SGC exo In.NC) were co-cultured with A-MSCs (**Figure 4H**). The miR-155 level in SGC7901 cell exosomes and A-MSCs treated with SGC exo In.miR-155 or SGC exo In.NC (100 µg/mL) for 8 h was measured by RT-PCR, which showed that it was significantly decreased in SGC exo In.miR-155 (*P* < 0.01) (**Figure 4I**) and A-MSCs treated with SGC exo In.miR-155 (*P* < 0.01) (**Figure 4J**). Under adipocyte differentiation conditions for 5 days, the expressions of C/EBPβ, C/EPBα, and PPARγ in A-MSCs co-cultured with SGC exo In.miR-155 (100 µg/mL) was much higher than that in the SGC exo In.NC group, **while** the expression of UCP1 was lower in SGC exo In.miR-155 group (*P* < 0.01) (**Figure 4L and 4M**). However, no significant change was observed in C/EBPβ mRNA in A-MSCs in these 2 groups (**Figure 5K**). Together, these results suggested that upregulation of GC exosomal miR-155 expression may have been responsible for the decreased expression level of C/EBPβ, thus inducing the trend of browning differentiation and poor adipogenesis in A-MSCs.

### The miR-155 contributes to the loss of adipose tissues *in vivo*

To further investigate the biological role of GC exosomal miR-155 in CAC, BALB/c nude mice were intravenously injected with exosomes derived from SGC7901 cells transfected with lentivirus containing a miR-155 overexpression (SGC7901-Exo miR-155 OE) sequence, miR-155 shRNA (SGC7901-Exo miR-155 KD), or control lentivirus (SGC7901-Exo). GES-1 exosomes (GES-1-Exo) were used as normal controls. The weight of the mice was reduced in the miR-155 OE group, but was increased in the SGC7901-Exo miR-155 KD group (*P* < 0.05) (**Figure 5B**). SGC7901exosomes stained with PKH67 were injected into the tail veins of mice for 24 h, followed by the detection of fluorescence signals in inguinal adipose tissue (**Figure 5C**). The level of miR-155 was enhanced in the inguinal adipose tissues and liver by GC exosomes, especially the GC exosomes with miR-155 overexpressed . The results are shown in **Supplementary Figure S3B**.

Images of inguinal adipose tissues from the 4 groups are shown in **Figure 5D**. The lengths (*P* < 0.01) (**Figure 5E**) and weights (*P* < 0.05) (**Supplementary Figure S3A**) of these adipose tissues in the SGC7901-Exo miR-155 OE group were decreased, and as expected, SGC7901-Exo miR-155 KO rescued these trends. Hematoxylin and eosin (H&E) staining of inguinal adipose tissues showed that treatment with GC exosomal miR-155 led to a significant increase in multinodular brown fat-like areas (**Figure 5G**). Additionally, we detected the expression of UCP1 in inguinal adipose tissues by immunohistochemistry (IHC). UCP1 upregulation was detected in the GC exosome-injected groups and showed a positive correlation with miR-155 levels (**Figure 5F**). Together, these results demonstrated that GC exosomes carrying miR-155 could be delivered to adipose tissue, leading to a decrease in adipose mass and promotion of brown adipose differentiation.

### GC exosomal miR-155 is responsible for CAC by targeting C/EBPβ *in vivo*

We detected the lipid formation capacity in mA-MSCs after culturing in adipogenic medium (**Supplementary Figure S5A**), and found that miR-155 in serum exosomes (**Supplementary Figure S5B**) and mA-MSCs (**Supplementary Figure S5E**) were distinctly upregulated in the SGC7901-Exo miR-155 OE group (*P* < 0.01). The expressions of C/EBPβ, C/EBPα, and PPARγ in the mA-MSCs of each group indicated a negative relationship with the miR-155 levels in serum exosomes, and the abundances of UCP1 showed a consistent trend with miR-155 levels (*P* < 0.05) (**Supplementary Figure S5C and S5D**). Together, these findings indicated that GC exosomes carrying miR-155 promoted CAC by suppressing C/EBPβ in A-MSCs, thus leading to impaired LD formation and brown adipose differentiation.

## Discussion

Continuous adipose tissue mass loss can result in devastating outcomes in CAC patients, which cannot be explained by reduced food intake alone^17^. Patients with CAC often present symptoms of emaciation without anorexia, and nutritional supplementation fails to reverse CAC^18^. Numerous deeper mechanistic studies have shown that the loss of adipose tissue precedes skeletal muscle loss in CAC; nevertheless, the underlying mechanisms of adipose tissue depletion in CAC remain poorly understood^19^. Recent studies implied that inflammatory and immunological factors such as interleukin-6 and tumor growth factor-β increased lipolysis^20,21^. Furthermore, researchers have focused their attention on changes in adipogenesis influenced by cancer^22^. LDs and mitochondria are therefore significantly involved in cellular lipid energy metabolism, and overconsumption of energy by cancer of adipose tissues is a crucial process in CAC. However, little is known about the effects of exosomes on the changed catabolism of adipocytes in CAC.

In addition, miR-155 has been confirmed as an “oncomiR” in various cancer types, including GC and breast cancer^23^. Importantly, recent work reported that miR-155 was overexpressed in T-cell lymphoma and chronic lymphocytic leukemia, and acted as a predictive biomarkers^24,25^. According to our clinical results, we confirmed that a high level of miR-155 expression was detected in the serum of GC patients, and that adipogenesis was blocked in A-MSCs obtained from GC patients. Notably, the levels of miR-155 in A-MSCs obtained from GC patients exhibited a negative relationship with the capacity of adipogenesis. These results led us to hypothesize that exosomal miR-155 may act as a promoting factor for CAC by attenuating adipogenesis of A-MSCs. As expected, miR-155 suppressed adipogenesis and increased brown adipose differentiation of A-MSCs. First, miR-155 was upregulated in the serum of GC patients and exosomes derived from GC cell lines. Second, adipogenesis was impaired by GC exosomes, which could have increased brown adipose differentiation of A-MSCs. Third, when mice were injected with GC exosomes overexpressing miR-155, the weight of the mice and the size of the inguinal adipose tissues were dramatically reduced, and UCP1 expression was high in adipocytes. It is therefore necessary to further evaluate the underlying mechanisms of how exosomal miR-155 affects the transdifferentiation of adipocytes.

Adipogenesis is a highly controlled process that occurs by sequential activation of transcription factors. C/EBPβ is rapidly (< 8 h) expressed during the induction of adipodifferentiation^26^. The C/EBPβ^-^/^-^ mice cells fail to undergo mitotic clonal expansion and differentiate into adipocytes. This finding provides further compelling proof that C/EBPβ is indispensable for adipogenesis^27^. Beginning 12 h after induction of adipodifferentiation, C/EBPβ transcriptionally activates the *C/EBP*α and *PPAR*γ genes^28^. Subsequently, C/EBPα and PPARγ function together to transactivate a large group of adipogenesis-associated genes, such as *FATP1, FABP4*, and *LPL*, which are essential for the formation of mature adipocytes^29,30^. *In vitro*, we confirmed that miR-155 inhibited the expression of C/EBPβ by binding sites in the 3′-UTR of C/EBPβ mRNA. Both C/EBPα and PPARγ, triggered by C/EBPβ, were also downregulated, while the brown adipose marker, UCP1, was upregulated. *In vivo*, the expressions of C/EBPβ, C/EBPα, and PPARγ in mA-MSCs were suppressed due to uptake of exosomal miR-155. Thus, these findings proved that GC exosomal miR-155 impaired adipogenesis of A-MSCs by inhibiting C/EBPβ. Moreover, exosomal miR-155 demonstrated bidirectional effects. UCP1 enrichment in mA-MSCs treated with exosomal miR-155 led to excessive energy expenditure.

In this study, we reported that miR-155 was upregulated in both the serum and A-MSCs of GC patients, and attenuated the adipogenesis of A-MSCs obtained from GC patients. We also found that GC exosomal miR-155 was internalized into A-MSCs, which inhibited adipogenesis progression of A-MSCs targeting C/EBPβ, inhibited C/EPBα and PPARγ, and induced brown adipocyte differentiation by upregulation of UCP1. In an animal model, knockdown of miR-155 in GC exosomes substantially reversed adipogenic-specific protein inhibition and overexpression of UCP1 in mA-MSCs. In conclusion, the present study provided insights into the significant role of miR-155 delivered by GC exosomes in reprogramming energy metabolism and promoting CAC. Adipogenesis and lipolysis are 2 important processes that maintain the balance of adipose tissue metabolism, but they are not completely independent. The effects of GC exosomes on adipose tissue include adipocyte differentiation and lipolysis. This study focused on adipocyte differentiation. However, the lipolysis of adipocytes has not been studied. Thus, we plan to study the influence of GC exosomes on the lipolysis of adipocytes to further determine the influences of GC exosomes on adipose tissues.

## Conclusions

The miR-155 was highly expressed in GC cells and could be extracellularly trafficked through exosomes. GC exosomal miR-155 inhibited adipogenesis in A-MSCs by targeting C/EBPβ and promoting browning differentiation of A-MSCs. *In vivo*, we confirmed that downregulation of miR-155 in exosomes inhibited the loss of fat volume and differentiation to brown adipocytes; thus, it can be used as a potential therapeutic target for CAC caused by GC (**Supplementary Figure S6**).
