# Supplementary material for: Exosomal miR-155 from gastric cancer induces cancer-associated cachexia by suppressing adipogenesis and promoting brown adipose differentiation via C/EPBβ
Source: Cancer Biol Med. 2022 Feb 19;19(9):1301–14. doi: 10.20892/j.issn.2095-3941.2021.0220 (PMC9500219; doi:10.20892/j.issn.2095-3941.2021.0220)
Supplement: Supplementary file 1 [file cbm-19-1301-s001.pdf]

Supplementary materials

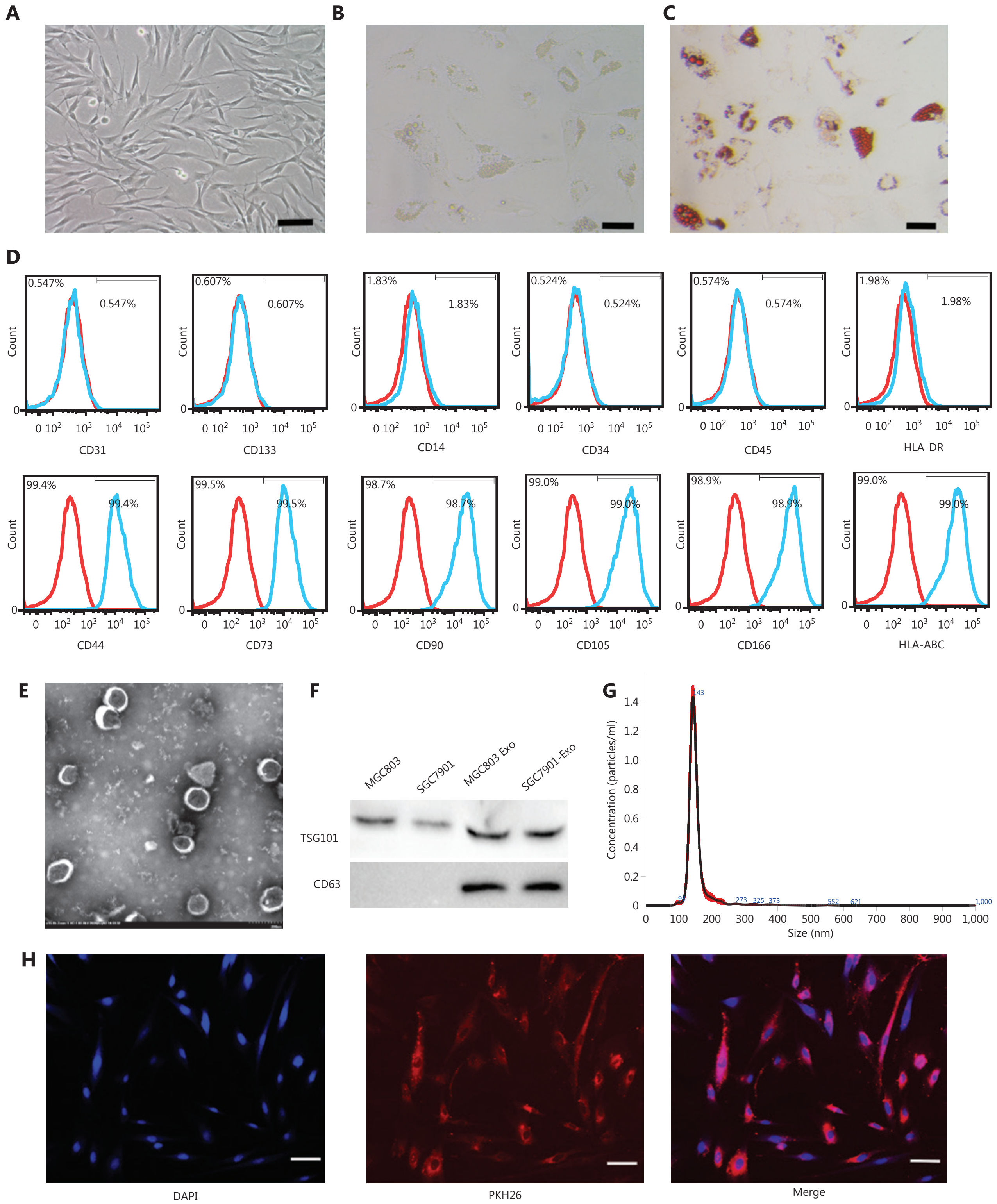

Figure S1 Identification and characterization of adipocyte differentiation of adipose mesenchymal stem cells (A-MSCs) and gastric cancer exosomes. (A) Morphology of A-MSCs. (B) Morphology of A-MSCs after adipodifferentiation. (C) Adipodifferentiation of A-MSCs stained with

Oil Red O. (D) Immunophenotypic analysis of A-MSCs. (E) Representative transmission electron microscopy image of exosomes derived from GC cells (scale bar = 200 nm). (F) Representative images of TGS101 and CD63 expression and exosome-specific markers. (G) Nanoparticle tracking analysis of SGC7901 exosomes. (H) PKH67-labeled SGC7901 exosomes can be taken up by A-MSCs.

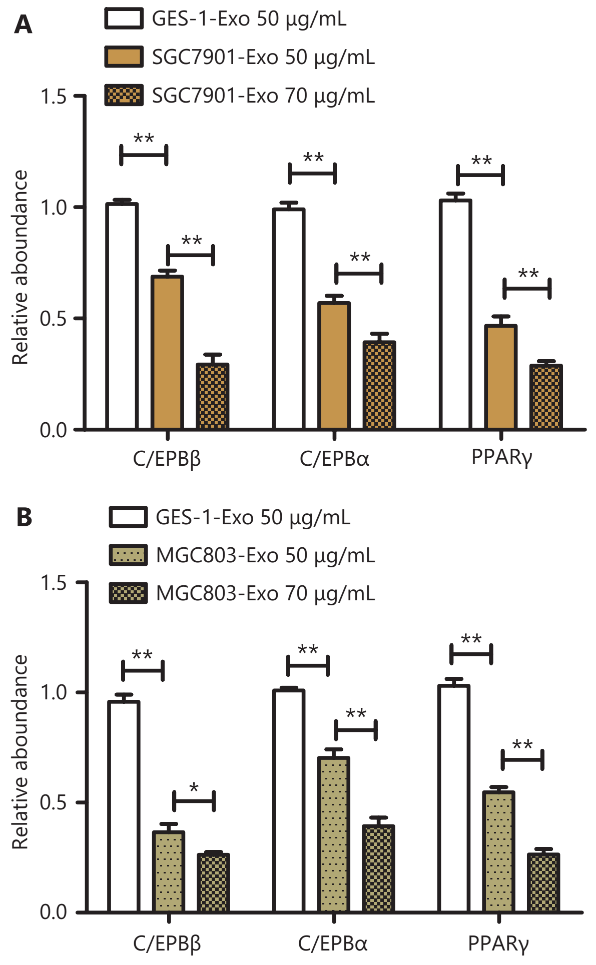

Figure S2 Densitometry analysis of C/EBPβ, C/EBPα, and PPARγ. (A) Densitometry analysis of C/EBPβ, C/EBPα, and PPARγ in the adipose mesenchymal stem cells (A-MSCs)+GES-1-Exo (50 μg/mL), A-MSCs+SGC-7901-Exo (50 μg/mL), and A-MSCs+SGC-7901-Exo (70 μg/mL) groups. (B) Densitometry analysis of the C/EBPβ, C/EBPα, and PPARγ in the A-MSCs+GES-1-Exo (50 μg/mL), A-MSCs+MGC-803-Exo (50 μg/mL), and A-MSCs+MGC-803-Exo (70 μg/mL) groups. *P < 0.05; **P < 0.01.

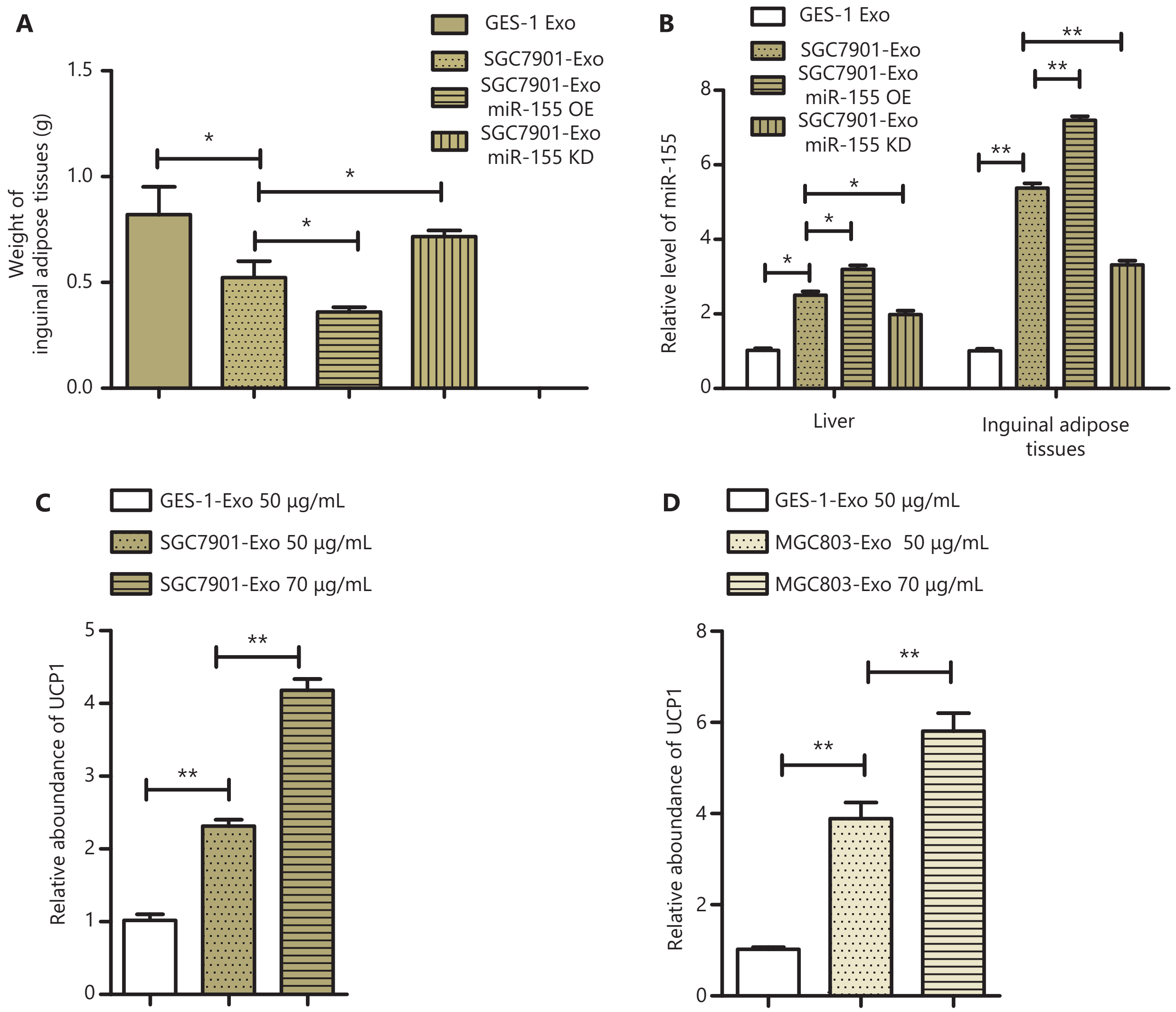

Figure S3 (A) Analysis of inguinal adipose tissue weights (*N* = 6). (B) RT-PCR assay of miR-155 levels in inguinal adipose tissues and the liver (*N* = 3). (C) Densitometry analysis of UCP1 in the adipose mesenchymal stem cells (A-MSCs)+GES-1-Exo (50 µg/mL), A-MSCs+SGC-7901-Exo (50 µg/mL), and A-MSCs+SGC-7901-Exo (70 µg/mL) groups. (D) Densitometry analysis of UCP1 in the A-MSCs+GES-1-Exo (50 µg/mL), A-MSCs+MGC-803-Exo (50 µg/mL), and A-MSCs+MGC-803-Exo (70 µg/mL) groups. *P < 0.05; **P < 0.01.

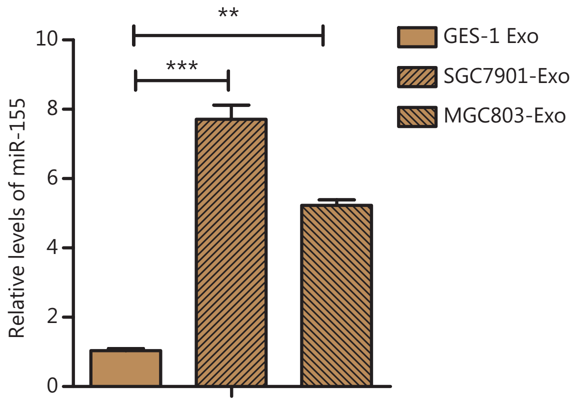

Figure S4 Relative levels of miR-155 in exosomes of gastric cancer cell lines (GES-1 exosomes, SGC7901 exosomes, or MGC803 exosomes) (*N* = 3). **P < 0.01; ***P < 0.001.

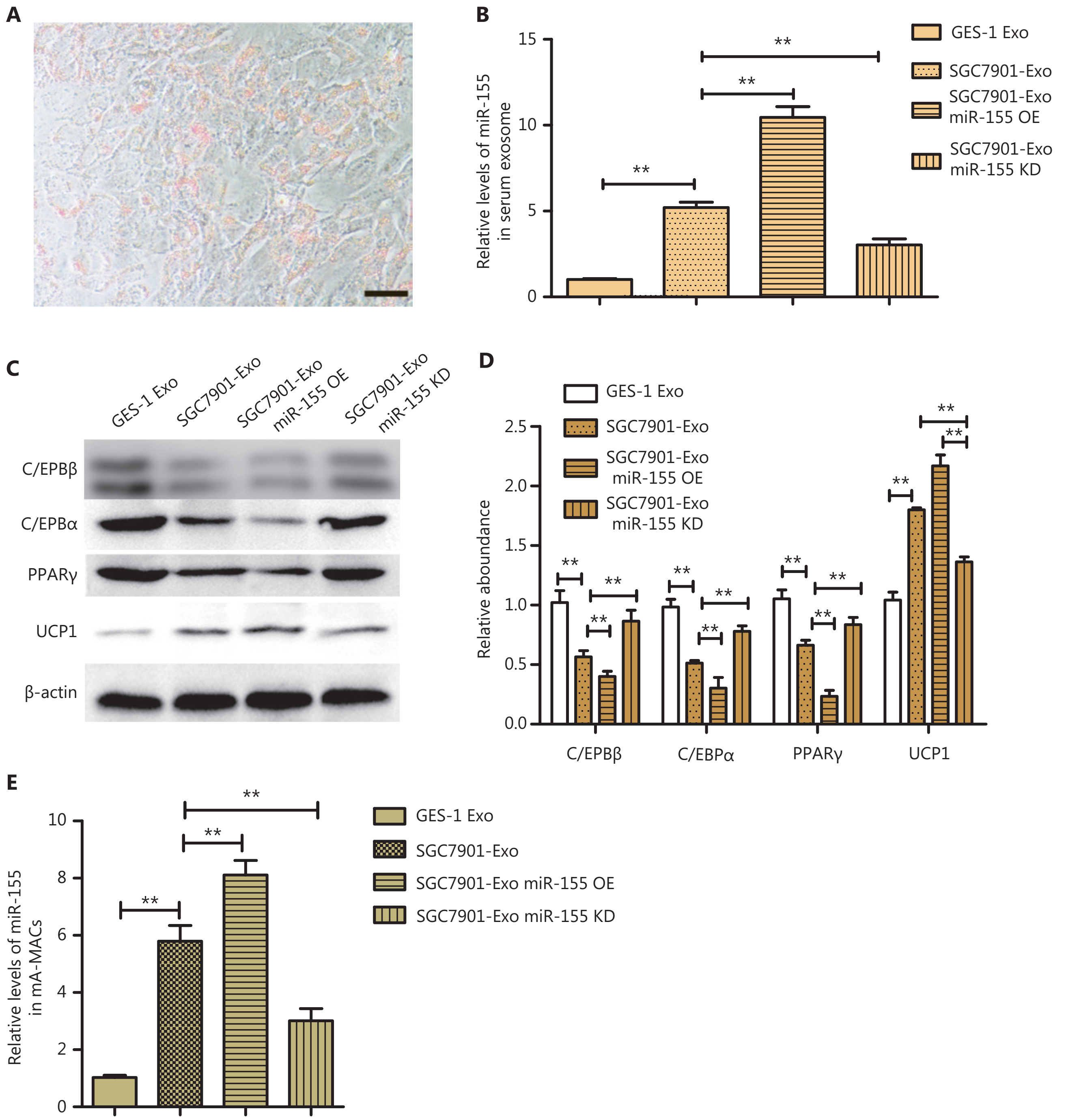

Figure S5 *In vivo* regulation of adipodifferentiation of gastric cancer exosomes in adipose mesenchymal stem cells (A-MSCs) by the miR-155/C/EBPβ axis. (A) The morphology of mA-MSCs after adipocyte differentiation (scale bar = 100 μm). (B) Relative levels of miR-155 in serum exosomes of mice (N = 3). (C–D) Western blot analyses of C/EBPβ, C/EBPα, PPARγ, and UCP1 in A-MSCs (N = 3). (E) Relative levels of miR-155 in mA-MSCs (N = 3). $^{**}P$ < 0.01.

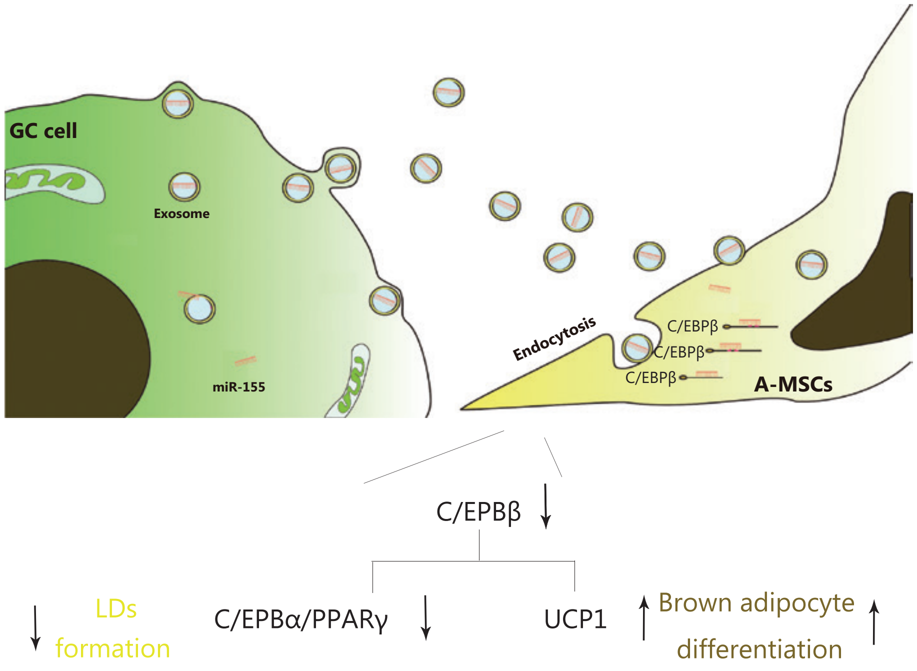

Figure S6 Schematic of conclusions.
